# Prevalence and Predictors of Impaired Coronary Flow Velocity Reserve in Adolescents After Arterial Switch Operation

**DOI:** 10.3390/diagnostics16070963

**Published:** 2026-03-24

**Authors:** Andrija Pavlovic, Milorad Tesic, Milan Djukic, Igor Stefanovic, Jasna Kalanj, Maja Bijelic, Maja Trkulja, Marko Pavlovic, Dusan Andric, Milica Kuzmanovic, Vladimir Milovanovic, Dejan Bisenic, Irena Ostric Pavlovic, Vojislav Parezanovic

**Affiliations:** 1 Cardiology Department, University Children’s Hospital, 11000 Belgrade, Serbiafloculus@gmail.com (M.T.); dusanandric97@gmail.com (D.A.);; 2 Medical Faculty, University of Belgrade, 11000 Belgrade, Serbiaanerio89@gmail.com (I.O.P.); 3 Cardiology Department, University Clinical Center of Serbia, 11000 Belgrade, Serbia; 4 Pediatric and Neonatal Intensive Care Unit, University Children’s Hospital, 11000 Belgrade, Serbia; 5 Cardiac Surgery Department, University Children’s Hospital, 11000 Belgrade, Serbia; 6 Clinical Immunology and Allergology Department, University Clinical Center of Serbia, 11000 Belgrade, Serbia

**Keywords:** coronary flow velocity reserve, D-transposition of the great arteries, arterial switch operation, adolescents, long-term follow-up

## Abstract

**Background/Objectives**: We assessed the prevalence of impaired coronary flow velocity reserve (CFVR) and aimed to identify echocardiographic and clinical predictors of coronary microvascular dysfunction in adolescents after neonatal arterial switch operation (ASO). **Methods**: This single-center, cross-sectional study included patients that underwent neonatal ASO for simple D-transposition of the great arteries (D-TGA) during 1998–2013. All patients were evaluated by echocardiography with global left ventricular strain measurement (GLS) and cardiac catheterization, including coronary angiography. Coronary flow velocity reserve was assessed by transthoracic Doppler echocardiography in the left anterior descending artery (LAD) using adenosine induced hyperemia. Patients were stratified into two groups according to CFVR: group with impaired CFVR (<2.5) and group with normal CFVR (≥2.5). Spearman correlation was used to assess the relationship between CFVR and echocardiographic variables. Binary logistic regression was used to determine independent predictors of impaired CFVR. **Results**: Out of 48 patients included (median age 16 years, age range 13 to 23 years, 71% male), impaired CFVR was found in 21 patients (44%). These patients had decreased longitudinal tricuspid annular plane systolic excursion (TAPSE), greater Z scores for left ventricular end-systolic dimensions and higher mean pulmonary artery pressures (mPAP). CFVR showed modest but significant positive correlations with tricuspid annular plane systolic excursion (TAPSE). Left pulmonary artery branch stenosis, reduced TAPSE and mPAP ≥ 20 mmHg, were significantly associated with impaired CFVR, while decreased TAPSE remained independent predictor in multivariable analysis (odds ratio 5.6, 95% confidence interval 1.24–25.26, *p* = 0.025). **Conclusions**: Impaired CFVR appears to be frequently observed in adolescents after uncomplicated neonatal ASO for simple D-TGA. Importantly, impaired CFVR is associated with right ventricular dysfunction.

## 1. Introduction

Dextro-transposition of the great arteries (D-TGA) is the most common cyanotic congenital heart disease in the neonatal period, accounting for 5–7% of all congenital heart defects and about one-third of conotruncal anomalies [1,2]. The arterial switch operation, since its introduction in 1976 by Adib Jatene, has become the standard surgical approach for D-TGA in most developed countries, offering excellent long-term outcomes, with survival rates of 93–97%, and operative mortality of 3–5% [3,4,5]. Consequently, an increasing number of patients are reaching adolescence and adulthood, shifting the focus from survival to long-term follow-up, identification and management of late sequelae.

Among the most relevant concerns during long-term follow-up after ASO are coronary complications [6,7,8,9]. Although coronary artery transfer can be successfully performed in all anatomical variants, somatic growth along with remodeling of the great arteries may predispose coronary arteries to kinking, compression, or scarring at reimplantation sites [7]. The incidence of late coronary obstruction is estimated at 3–8%. Furthermore, a substantial number of cases remains clinically silent, due to inevitable myocardial denervation and consequential masking of the ischemic symptoms [4,6,7,8,9].

Contemporary guidelines recommend both multi-slice computed tomography (MSCT) coronary angiography as well as selective coronary angiography as a standard diagnostic tools for evaluation of coronary artery stenosis in presence of electrocardiographic (ECG) or echocardiographic signs of myocardial ischemia, or in the presence of complex coronary anatomy [6]. While both methods demonstrate high sensitivity and specificity for detecting lesions in the large epicardial vessels, they provide little information on the coronary microcirculation and microvascular function. Cardiac magnetic resonance (CMR) perfusion imaging has been utilized as a non-invasive tool with good results in pediatric and young adult populations, though its use remains limited by restricted availability [10,11].

Given these limitations, there is a growing need for reliable functional assessment of coronary circulation beyond the epicardial vessels. Coronary arterioles and capillary vessels comprise nearly 75% of entire coronary circulatory bed [12]. Coronary flow velocity reserve (CFVR), measured non-invasively by transthoracic Doppler echocardiography in the left anterior descending artery during pharmacologic hyperemia, provides valuable insight into coronary microvascular function [12,13]. Unlike MSCT or conventional angiography, which primarily evaluate large-vessel anatomy, CFVR reflects the integrity of the microcirculatory bed and has emerged as a sensitive marker of early coronary dysfunction. However, data on CFVR in patients after ASO remain scarce, particularly in adolescents and young adults, despite their increasing clinical relevance [14]. Recent systematic reviews have highlighted that coronary microvascular dysfunction in congenital heart disease remains incompletely understood, with heterogeneous methodologies and limited data regarding its prevalence and clinical relevance in pediatric populations [15].

Therefore, we sought to evaluate the prevalence of impaired coronary flow velocity reserve in adolescents after ASO and to identify clinical and echocardiographic predictors of coronary microvascular dysfunction in this population.

## 2. Materials and Methods

### 2.1. Study Design and Population

This was a single-center, cross-sectional, observational cohort study, which included a cohort of patients who previously underwent neonatal ASO for simple D-TGA in the cardiac surgery department at University Children’s Hospital in Belgrade, Serbia, between 1998 and 2013. The patient data regarding ASO were retrieved from the electronic database of the cardiac surgery department.

### 2.2. Exclusion and Inclusion Criteria

Eligible patients have met the following criteria: (1) neonatal ASO for simple D-TGA, (2) Age at follow-up ≥ 12 years, (3) asymptomatic at the time of inclusion. Exclusion criteria were as follows: (1) complex D-TGA, (2) surgical technique other than ASO for treatment of D-TGA, (3) previous catheter and/or surgical reintervention after neonatal ASO, (4) known coronary artery complications in the follow-up. Complex D-TGA was defined as D-TGA associated with ventricular septal defect (VSD), left ventricular outflow tract obstruction (LVOTO), coarctation of the aorta (CoAo), interrupted aortic arch, or other significant intracardiac anomalies. This study was conducted in accordance with the Declaration of Helsinki. Prior to initiation of the study, the University Children’s Hospital Ethics Committee provided approval (reference no. 017/02, approval date: 2 December 2019).

### 2.3. Baseline Clinical Assessment

All patients underwent ASO for simple D-TGA at neonatal age. At the time of evaluation, demographic characteristics, detailed previous medical history and physical examination including anthropometrics has been recorded. N-terminal pro-B-type natriuretic peptide (NTproBNP) levels were measured at admission (ECLIA, Roche diagnostics, Basel, Switzerland), and Zlog value was calculated according to age [16]. Standard 12-lead electrocardiogram (ECG) and chest X-ray were performed for every patient at admission. Additionally, prior to or after hospitalization, all patients underwent a 24 h ECG Holter monitoring and standardized exercise stress test following modified Bruce protocol in order to exclude arrhythmia or exercise induced myocardial ischemia [17].

### 2.4. Echocardiographic Assessment

Standard two-dimensional (2D) and Doppler transthoracic echocardiography was performed in all patients at admission, using a standardized imaging protocol in accordance with contemporary pediatric and adult congenital echocardiography guidelines [10,18]. All studies were conducted by experienced pediatric cardiologist using a Vivid E95 ultrasound system (GE HealthCare, Chicago, IL, USA). All measurements represent the average of three consecutive cardiac cycles. Images were acquired using standard two-dimensional, M-mode, Doppler techniques in subcostal, apical, parasternal and suprasternal views with ECG gating. Measurements included left ventricular end-diastolic (LVEDD), end-systolic diameters (LVESD), interventricular septal and posterior wall thickness, left atrial end-systolic diameter. Atrioventricular valves were measured from 4-chamber apical view and were assessed by color Doppler techniques. Morphometric analysis of the neoaorta and neopulmonary artery was performed from the parasternal long-axis view [18]. Acquired dimensions were standardized using the corresponding Z scores. Neoaortic and neopulmonary stenosis or dilation was determined as a Z score ≥ 2, respectively [19]. Left ventricular systolic function was assessed by linear measurement of shortening fraction (SF) in parasternal short-axis M-mode, mitral annular plane systolic excursion (MAPSE) and volumetric measurement of ejection fraction (LVEF) via modified biplane Simpson method. Right ventricular function was assessed by tricuspid annular plane systolic excursion (TAPSE), measured by M-mode at the tricuspid valve free wall annulus from the 4-chamber, apical view [18,20]. Right ventricular systolic pressure was assessed from the peak tricuspid valve regurgitation velocity, calculated by modified Bernoulli equation, with addition of estimated right atrial pressure. Elevated right ventricular systolic pressure (RVSP) was defined as >35 mmHg [20,21,22,23]. Early (E) and late (A) diastolic mitral and tricuspid inflow velocities were measured using pulsed-wave Doppler at the level of the mitral and tricuspid leaflet tips, respectively, in accordance with published pediatric normative data [24,25].

### 2.5. Left Ventricular Global Longitudinal Strain (GLS) Analysis

In addition to a standard 2D echocardiographic assessment, all patients underwent a left ventricular global longitudinal strain (GLS) assessment in concordance with contemporary guidelines [18,26,27,28]. Two-dimensional speckle tracking echocardiography (2D-STE) was used for left ventricular deformation analysis. Minimum three consecutive cardiac cycles, with ECG gating were recorded and stored in cine-loop format, with frame rates of 70 to 90 Hz [26]. The acquisitions were performed in an apical 3-chamber (or long-axis), 4-chamber and 2-chamber views, with clear visualization and manual tracing of endocardial borders in the end-systolic frame. Aortic valve closure timing was automated and verified manually by experienced echocardiographer. Then, GLS was calculated offline using vendor specific software (EchoPAC, version 201, GE HealthCare, Chicago, IL, USA); endocardial borders were manually traced at end-systole, after which automated speckle-tracking was performed with manual adjustments when needed to optimize segmental tracking. The left ventricle was divided into six segments in each view, and GLS was obtained as the average longitudinal strain from all segments and reported as an absolute value (%) [25,27]. Normal GLS was determined according to contemporary pediatric normative values [27,29].

### 2.6. Coronary Flow Velocity Reserve Assessment

Coronary flow velocity reserve was assessed non-invasively by transthoracic Doppler echocardiography. A special preset for coronary flow velocity assessment was provided by the vendor, on a 4 MHz transducer. A color Doppler flow map was optimized, by setting the Nyquist limit to 16–24 cm/s. The distal segment of the left anterior descending coronary artery (LAD) was visualized in a modified apical 4-chamber view, and rotated until coronary artery flow was identified by color Doppler. Pulsed-wave Doppler imaging was then applied with a 3 to 5 mm sample volume, ensuring the ultrasound beam was aligned as parallel as possible to coronary flow, and as stable as possible during measurements. Peak diastolic flow velocity was measured at the baseline and during pharmacologically induced hyperemia. Maximum hyperemia was achieved by intravenous adenosine infusion at 140 µg/kg/min for 2 min, with continuous ECG and blood pressure monitoring. CFVR was calculated as the ratio of hyperemic to resting peak diastolic flow velocity [30,31,32]. Patients with CFVR < 2.5 were defined as having impaired coronary flow reserve, in accordance with prior transthoracic Doppler studies demonstrating prognostic relevance of this threshold in adult and mixed cardiovascular populations [32,33]. Although CFVR is inherently a continuous variable, dichotomization was performed to facilitate clinically interpretable group comparisons and regression modeling, in line with prior prognostic CFVR studies. Although pediatric-specific validation of CFVR thresholds remains limited, Doppler-based assessment of coronary flow and coronary flow reserve has been applied in pediatric congenital heart disease populations [34,35]. Therefore, this cut-off was adopted for consistency with established CFVR research, while acknowledging the need for age-specific validation. Accordingly, in this adolescent congenital heart disease cohort, the <2.5 threshold should be considered exploratory and used primarily for clinically interpretable stratification. Therefore, our findings should not be interpreted as validating a pediatric-specific CFVR threshold, but rather as exploratory signal-generating data.

### 2.7. Cardiac Catheterization

All patients in the cohort underwent diagnostic cardiac catheterization, according to the standardized protocol for adolescent patients after ASO during long-term follow-up at our institution [6]. The procedure was performed in accordance with contemporary international expert consensus recommendations for cardiac catheterization in congenital heart disease, including standards for anatomic imaging, hemodynamic assessment, and radiation-safety practice [36]. Catheterizations were performed via transfemoral arterial and venous approach, respectively. After vascular access was obtained, a left-sided cardiac catheterization was performed. Aortography was carried out to visualize supravalvular stenosis or aortic dilation. Coronary angiography was done in all patients to exclude significant epicardial coronary artery stenosis, which was defined as ≥50% of luminal narrowing in any major epicardial artery [37]. Subsequently, right-sided catheterization, with right ventriculography and pulmonary angiography was performed to assess the position and potential stenosis of the pulmonary artery branches after LeCompte maneuver. Direct hemodynamic pressures were recorded as the mean of three consecutive measurement in the aorta, left ventricle, right atrium, right ventricle, pulmonary artery and pulmonary artery branches. Pulmonary artery branch stenosis was defined as ≥30% luminal diameter reduction on angiography and/or a systolic pressure gradient ≥ 20 mmHg across the stenotic segment, according to contemporary guidelines [38].

### 2.8. Statistical Analysis

Statistical analysis was performed using SPSS Statistics version 23 (SPSS, Chicago, IL, USA). A two-sided *p* value < 0.05 was considered statistically significant. Descriptive data were expressed as means with standard deviation for continuous variables, and as counts with percentage for categorical variables. The normality of the data was assessed by Shapiro–Wilk test. Differences between continuous variables were compared using Student’s *t*-test or Mann–Whitney U test, according to data distribution, while χ^2^ test was used for categorical variables. Associations between coronary flow velocity reserve (CFVR) and clinical, echocardiographic, and invasive hemodynamic parameters were explored using Spearman rank correlation analysis. To identify predictors of impaired CFVR, binary logistic regression analysis was performed. In the univariable model, impaired CFVR was entered as the dependent variable and tested against individual explanatory variables, including demographic characteristics, perioperative parameters, long-term postoperative sequelae, echocardiographic measures, NT-proBNP levels, and catheterization findings. Variables demonstrating statistical significance in univariable analysis (*p* < 0.05) were subsequently entered into a multivariable logistic regression model to identify independent predictors of impaired CFVR.

## 3. Results

### 3.1. Study Population and Baseline Characteristics

**Figure 1:**
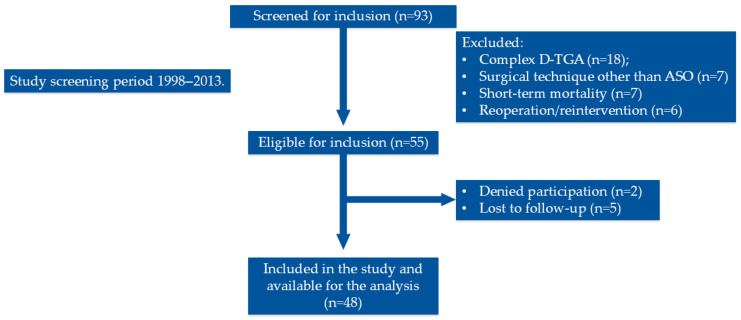
Study flow-chart.

During the period from 1998 to 2013, a total of 93 patients were treated for D-TGA at University Children’s Hospital. After exclusion, 48 patients who underwent ASO for simple D-TGA at neonatal age were included in the study (Figure 1). Baseline characteristics of the cohort are presented in Table 1. The mean age was 16.0 ± 2.8, and patients were predominantly male (71%). Mean age at the time of surgery was 19 ± 10 days, and D-TGA was prenatally recognized in 15% of patients. The majority of patients received continuous intravenous prostaglandin E1 (PGE1) infusion prior to surgery, and balloon atrial septostomy (the Rahskind procedure) has been performed in 85% of the patients prior to surgery. There were no statistically significant differences between groups with preserved and impaired CFVR in regard to sex, age, NTproBNP levels, and preoperative, intraoperative and postoperative parameters.

### 3.2. Echocardiographic Parameters and Coronary Flow Velocity Reserve

Echocardiographic findings for the entire cohort, as well as groups with preserved and impaired CFVR are summarized in Table 2. All patients underwent successful CFVR measurement. In this selected cohort of asymptomatic adolescents after uncomplicated neonatal ASO for simple D-TGA (excluding patients with prior reinterventions or known coronary complications), impaired CFVR was identified in 21 patients (44%), while 27 patients (56%) demonstrated preserved CFVR.

All patients had preserved LVEF. However, reduced longitudinal systolic indices were frequently observed: MAPSE and TAPSE were reduced in almost 30% and 44% of patients, respectively. Left ventricular GLS values were below age-adjusted reference limits in approximately one third of the cohort. Neoaortic root dilation was present in 79% of patients.

When comparing patients with impaired and preserved CFVR, those with impaired CFVR had higher LVESD Z scores, while the prevalence of reduced TAPSE was significantly higher. Although absolute TAPSE values did not reach statistical significance (*p* = 0.064), the prevalence of reduced TAPSE was significantly higher in the impaired CFVR group (*p* = 0.040). There was no statistically significant difference between groups regarding LVEF, SF, cardiac dimensions with corresponding Z scores, and GLS values.

### 3.3. Catheterization Findings

Invasive hemodynamic assessment and cardiac catheterization findings are presented in Table 3. No patients had significant epicardial coronary stenosis. Mean pulmonary artery pressure was mildly but significantly higher in patients with impaired CFVR compared to those with preserved CFVR (*p* < 0.05). Furthermore, left pulmonary artery branch stenosis was more frequent in the impaired CFVR group (*p* = 0.051). There were no statistically significant differences between in other pressure measurements between groups.

### 3.4. Correlation Analysis and Binary Logistic Regression

**Figure 2:**
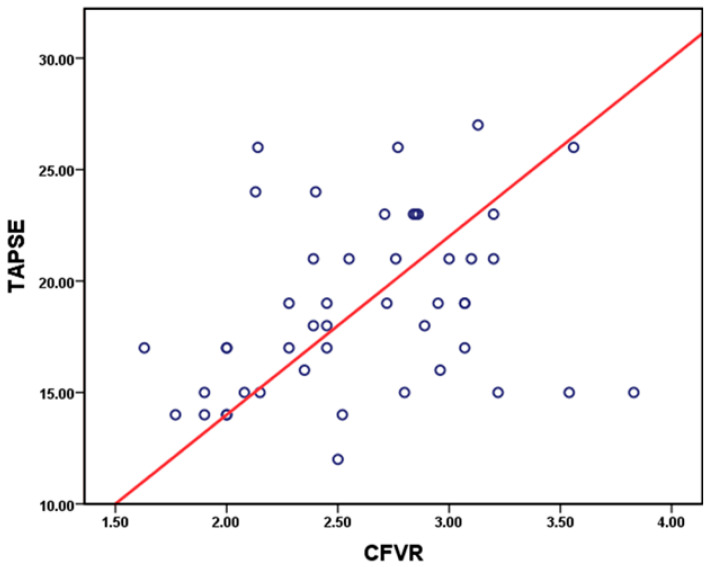
Scatter plot demonstrating the relationship between TAPSE and CFVR in the study cohort. Circles represent individual patients, and the red line represents the linear regression line (r = 0.35, *p* = 0.016).

Spearman correlation demonstrated modest, but statistically significant, positive correlation between TAPSE and CFVR (r = 0.35, *p* = 0.016). Correlation scatter plot is presented in Figure 2. When analyzing other variables, there was no statistically significant correlation with CFVR.

To identify independent predictors of reduced CFVR, binary logistic regression was performed. In univariable analysis, left pulmonary artery branch stenosis (odds ratio (OR) 5.92, 95% confidence interval (CI) 1.06–32.89, *p* = 0.042), reduced TAPSE (OR 3.86, 95% CI 1.15–12.91, *p* = 0.028) and mPAP ≥ 20 mmHg (OR 3.64, 95% CI 1.04–12.78, *p* = 0.043), were significantly associated with impaired CFVR. In multivariable analysis, reduced TAPSE was independently associated with impaired CFVR (OR 5.60, 95% CI 1.24–25.26, *p* = 0.025). Mean pulmonary artery pressure ≥ 20 mmHg and left pulmonary artery branch stenosis were associated with impaired CFVR in univariable analysis but did not retain independent significance after adjustment.

## 4. Discussion

In the present study, there are two main findings. The first finding reveals a substantial proportion of patients with impaired coronary flow velocity reserve (44%), in a well-characterized, contemporary cohort of adolescent patients after neonatal arterial switch operation for simple D-TGA, despite preserved epicardial coronary anatomy and global systolic function. Importantly, this prevalence should be interpreted within the context of our inclusion criteria: we intentionally studied a homogeneous subgroup with simple D-TGA after neonatal ASO, without prior surgical/catheter reinterventions and without known coronary complications. Therefore, the observed prevalence reflects coronary microvascular impairment in a relatively low-risk, asymptomatic post-ASO population and may not be directly generalizable to the broader, unselected ASO population. Secondly, reduced CFVR was associated with indices of right ventricular dysfunction, and elevated pulmonary artery pressures, suggesting a potential link between right ventricular function and microvascular dysfunction in this group of patients.

Majority of previous studies have focused on anatomical epicardial coronary artery stenosis as a late sequalae after neonatal ASO, while functional microvascular impairment has received less attention [4,5,7,9]. Coronary microvascular dysfunction in children has increasingly been recognized as a distinct and underexplored pathological entity, with potential implications for long-term myocardial remodeling and cardiovascular risk stratification in congenital heart disease populations [39]. To our knowledge, this is the largest cohort of patients with simple D-TGA treated with ASO at neonatal age assessed for coronary flow velocity reserve [14,40,41]. The observed prevalence of impaired CFVR in our cohort is notably higher, than in previous invasive Doppler and angiographic study by Oscarsson et al. [40]. This might be attributed to differences between invasive and non-invasive CFVR assessment. Likewise, studies by Gagliardi and Hauser, have confirmed significantly reduced CFVR in patients after coronary artery transfer, respectively [14,41]. These findings are similar to our results, and support the concept of abnormal coronary vasomotor response in D-TGA patients in the long-term follow-up after ASO, even in the absence of fixed epicardial coronary stenosis. Moreover, all patients in the present study were clinically asymptomatic, had preserved left ventricular ejection fraction and normal coronary angiograms. This underscores the limited sensitivity of conventional clinical and anatomical assessments in early detection of microvascular dysfunction in the post-ASO cohort.

There are several factors contributing to impaired CFVR after ASO. Of note, during ASO, surgical coronary transfer inevitably damages cardiac autonomic plexus, resulting in partial or complete coronary denervation. This leads to impaired vasomotor response and changes in coronary flow regulation [9,42]. Additionally, progressive somatic growth and spatial remodeling of the great vessels, may result in alterations in coronary geometry, as well as in stretching or kinking. These changes might not result in hemodynamically significant coronary stenosis, but may alter coronary flow dynamics [8]. Lastly, endothelial dysfunction as a part of innate disease might be involved, unlike in populations with history of complete heart denervation, such as in heart transplant patients [43,44]. Combined, these mechanisms may provide a possible explanation for the discrepancy between preserved epicardial coronary anatomy and impaired coronary microvascular function in significant portion of our cohort.

An important and novel observation in our study is the association between impaired CFVR and markers of right ventricular dysfunction, particularly reduced TAPSE, higher mean pulmonary artery pressures and higher frequency of left pulmonary artery branch stenosis. Although these variables did not all maintain independent significance in multivariable analysis, their association with impaired CFVR suggests a pathophysiological interplay between right ventricular performance, pulmonary artery pressure and coronary microvascular function.

Ventricular interdependence may represent a potential mechanistic link between right ventricular dysfunction and coronary microvascular function [45,46]. Increased right ventricular afterload may lead to septal displacement and alterations in left ventricular geometry, potentially contributing to impaired diastolic filling and increased myocardial wall stress [46]. These changes could adversely influence coronary microvascular reserve, even in the absence of significant epicardial coronary stenosis. Furthermore, an association between right ventricular pressure overload and coronary microvascular dysfunction has been described in previous studies [22,45,47,48], although the causal relationship remains incompletely understood.

In patients after ASO, a common sequela, occurring in up to 42% of patients in the long-term follow-up is pulmonary artery branch stenosis, following LeCompte maneuver [4,10,49]. Therefore, despite ASO restoring normal ventriculo-arterial connection, subtle changes in the right ventricular function and pulmonary circulation may persist. Although pulmonary artery branch stenosis did not remain an independent predictor in multivariable analysis, its association with impaired CFVR in univariable analysis suggests that chronic right ventricular pressure overload may contribute to coronary microvascular dysfunction. However, the observed differences in mPAP were modest and likely reflect borderline hemodynamic variation; therefore, this association should be interpreted conservatively. Importantly, right ventricular systolic function, assessed by TAPSE, emerged as the only independent predictor of impaired CFVR in multivariable analysis. This finding suggests that right ventricular performance may represent a potential integrative marker of cardiopulmonary interaction and coronary microvascular function in this population.

Interestingly, although left ventricular GLS was reduced in approximately one third of the cohort, GLS values did not differ significantly between groups with impaired and preserved CFVR. Furthermore, we did not find statistically significant association between CFVR and GLS in our group of patients. Several adult studies have demonstrated a significant association between impaired coronary flow reserve and reduced left ventricular global longitudinal strain, suggesting that microvascular dysfunction may precede or accompany subtle systolic impairment detectable by deformation imaging. In contrast, other investigations have failed to identify a meaningful correlation between CFR and GLS, particularly in cohorts with preserved left ventricular ejection fraction and absence of overt ischemic heart disease [50,51,52]. Our findings indicate that subclinical left ventricular dysfunction may be present in adolescents after ASO, but it does not appear to be primary determinant of microvascular impairment at this stage of follow-up. In contrast, TAPSE demonstrated a closer relationship with CFVR, highlighting the dominant role of right-sided cardiopulmonary interactions in the pathophysiology of microvascular dysfunction in this population. Furthermore, these findings indicate that coronary microvascular dysfunction may precede or occur independently of overt left ventricular deformation abnormalities.

There are several limitations to our study. Firstly, selection bias should be acknowledged. By design, we excluded patients with complex D-TGA, prior catheter/surgical reinterventions, and known coronary complications. While this improved cohort homogeneity, it limits external validity; the true prevalence of impaired CFVR in an unselected ASO population—particularly among patients with complex anatomy or prior coronary events—may differ (and could be higher). Secondly, this is a single-center, cross-sectional study with a relatively small sample size, which may limit statistical power and contribute to wide confidence intervals, particularly in multivariable analyses. Due to the observational and exploratory nature of the study, a formal a priori sample size calculation was not performed. The cohort included all eligible patients within the predefined study period. The limited number of events may have affected the stability of the multivariable model, increased the risk of overfitting and contributed to imprecision in the estimated effect sizes. Therefore, the independent association between reduced TAPSE and impaired CFVR should be interpreted as hypothesis-generating and requires confirmation in larger multicenter cohorts. Coronary flow velocity reserve was assessed non-invasively by transthoracic Doppler echocardiography, rather than by invasive coronary flow measurements or positron emission tomography. Although transthoracic CFVR is a well-validated and widely used method, it remains operator-dependent and limited to assessment of the left anterior descending artery. In addition, the CFVR cut-off of <2.5 was derived from adult- and mixed-population studies, and pediatric-specific validation in adolescents with congenital heart disease remains limited. Third, myocardial deformation analysis was limited to left ventricular global longitudinal strain, while advanced indices such as myocardial work were not assessed. Additionally, right ventricular assessment was primarily based on TAPSE, a load-dependent parameter of longitudinal RV function. Advanced right ventricular deformation indices, such as RV strain analysis, were not systematically evaluated. This represents an important limitation, as RV strain parameters may provide a more sensitive and comprehensive assessment of right ventricular mechanics and could have offered additional mechanistic insight into the relationship between RV performance and coronary microvascular reserve.

In conclusion, impaired CFVR appears to be frequently observed in adolescents after uncomplicated neonatal ASO for simple D-TGA, despite preserved epicardial coronary anatomy and global left ventricular systolic function. In this selected, asymptomatic cohort, impaired CFVR was associated with markers of right ventricular dysfunction and elevated pulmonary artery pressures, suggesting a potential interaction between pulmonary circulation, right ventricular performance, and coronary microvascular function. These findings support the concept that coronary microvascular dysfunction may represent an underrecognized component of late sequelae after ASO. However, given the cross-sectional design and modest sample size, the observed associations should be interpreted cautiously and require confirmation in larger, longitudinal multicenter studies. Functional assessment of coronary microcirculation and right ventricular performance may provide additional insight during long-term follow-up of post-ASO patients.

## Figures and Tables

**Table 1:** Baseline clinical and ASO characteristics in all patients, and in groups with impaired and preserved CFVR, respectively.

	All Patients (*n* = 48)	Impaired CFVR (*n* = 21)	Preserved CFVR (*n* = 27)	*p* Value
Age (years, mean, SD)	16.0 ± 2.8	16.3 ± 2.8	15.7 ± 2.8	0.47
Sex (male, %)	71.0	71.0	70	0.99
Weight (kg, mean, SD)	64.16 ± 23.54	66.18 ± 30.1	62.58 ± 17.27	0.34
BMI (kg/m^2^, mean, SD)	20.84 ± 3.27	20.49 ± 3.24	21.11 ± 3.33	0.52
BSA (m^2^, mean, SD)	1.70 ± 0.26	1.68 ± 0.24	1.71 ± 0.29	0.73
NTproBNP (pg/mL, mean, SD)	61.7 ± 60.2	48.2 ± 26.0	71.7 ± 68.2	0.27
Zlog NTproBNP (mean, SD)	0.45 ± 0.74	0.28 ± 0.64	0.57 ± 0.79	0.23
Preoperative parameters				
Age at surgery (days, mean, SD)	19 ± 10	17 ± 8	20 ± 11	0.28
Prenatal diagnosis (%)	15.0	18.0	9.0	0.44
Birth BW (kg, mean, SD)	3.32 ± 0.4	3.23 ± 0.4	3.36 ± 0.4	0.24
BW less than 3 kg (%)	13.0	19.0	7.0	0.38
Preoperative PGE1 infusion (%)	79.0	71.0	85.0	0.29
Rashkind procedure (%)	85.0	81.0	89.0	0.68
Aorta/Pulmonary artery ratio (mean, SD)	1.03 ± 0.35	1.11 ± 0.51	0.98 ± 0.13	0.22
Intraoperative and immediate postoperative characteristics				
Single coronary artery (%)	12.0	9.0	15.0	0.68
Left atrial pressure (mmHg, mean, SD)	10.6 ± 3.5	10.4 ± 2.8	10.8 ± 4.2	0.72
Central venous pressure (mmHg, mean, SD)	8.3 ± 2.6	7.8 ± 2.4	8.7 ± 2.8	0.37
MAP (mmHg, mean, SD)	47 ± 7.6	46.4 ± 5.3	47.7 ± 9.4	0.64
Time from operation to extubating (days, mean, SD)	4.0 ± 2.3	4.4 ± 2.1	4.7 ± 2.5	0.62
Low cardiac output syndrome (%)	22.0	16.0	27.0	0.48
Junctional ectopic tachycardia (%)	26.0	20.0	31.0	0.51
Peritoneal dialysis (%)	26.0	15.0	35.0	0.18
Diaphragm paresis (%)	4.0	3.0	5.0	0.99
Bleeding (%)	11.0	5.0	15.0	0.37
Myocardial ischemia (%)	13.0	5.0	18.0	0.22
Atelectasis (%)	9.0	8.0	10.0	0.99
Pneumothorax (%)	11.0	15.0	8.0	0.64
Any postoperative complication (%)	50.0	40.0	58.0	0.37

**Table 2:** Echocardiographic findings in all patients, and in groups with impaired and preserved CFVR, respectively.

	All Patients (*n* = 48)	Impaired CFVR (*n* = 21)	Preserved CFVR (*n* = 27)	*p* Value
Left atrium (mm, mean, SD)	26.5 ± 3.0	26.6 ± 3.0	26.4 ± 3.0	0.88
Left atrium Z score (mean, SD)	−0.16 ± 0.89	−0.13 ± 0.87	−0.19 ± 0.91	0.81
MV annulus (mm, mean, SD)	27.8 ± 3.8	27.4 ± 3.1	28.2 ± 4.4	0.46
MV annulus Z score (mean, SD)	−0.51 ± 0.90	−0.53 ± 0.84	−0.49 ± 0.96	0.87
TV annulus (mm, mean, SD)	27.5 ± 3.7	26.7 ± 4.0	28.1 ± 3.4	0.18
TV annulus Z score (mean, SD)	−0.58 ± 0.71	−0.60 ± 0.85	−0.56 ± 0.60	0.84
LVEDD (mm, mean, SD)	48.5 ± 5.2	48.0 ± 5.4	48.9 ± 5.0	0.54
LVEDD Z score (mean, SD)	−0.20 ± 1.00	−0.20 ± 1.03	−0.20 ± 1.00	0.99
LVESD (mm, mean, SD)	28.5 ± 5.6	28.6 ± 7.2	28.4 ± 4.1	0.86
LVESD Z score (mean, SD)	−0.47 ± 1.07	−0.13 ± 1.06	−0.74 ± 1.02	0.050
Shortening fraction (%, mean, SD)	40.0 ± 0.5	38.5 ± 0.4	42.2 ± 0.5	0.10
LV ejection fraction (%, mean, SD)	59.0 ± 4.5	58.7 ± 4.7	59.3 ± 4.5	0.65
Mitral valve E/A ratio (mean, SD)	1.71 ± 0.26	1.67 ± 0.22	1.74 ± 0.29	0.36
MAPSE (mm, mean, SD)	16.9 ± 2.5	17.4 ± 2.5	16.6 ± 2.5	0.28
Reduced MAPSE (%)	29.0	28.0	29.0	0.99
Tricuspid valve E/A ratio (mean, SD)	1.57 ± 0.31	1.53 ± 0.27	1.60 ± 0.34	0.39
TAPSE (mm, mean, SD)	18.8 ± 3.8	17.6 ± 3.5	19.7 ± 3.9	0.064
Reduced TAPSE (%)	44.0	62.0	30.0	0.040
Aorta (mm, mean, SD)	24.4 ± 3.0	24.2 ± 2.9	24.7 ± 3.1	0.59
Aorta Z score (mean, SD)	2.26 ± 1.06	2.29 ± 1.05	2.24 ± 1.09	0.87
Aortic dilation (%)	79.0	80.0	78.0	0.99
Pulmonary artery (mm, mean, SD)	22.0 ± 2.4	21.9 ± 2.3	22.2 ± 2.6	0.69
Pulmonary artery Z score (mean, SD)	−0.70 ± 0.74	−0.69 ± 0.81	−0.71 ± 0.70	0.93
Global longitudinal LV strain (%, mean, SD)	−19.3 ± 2.7	−18.9 ± 2.9	−19.5 ± 2.5	0.43
Reduced GLS (%)	29.0	29.0	30.0	0.99

**Table 3:** Invasive hemodynamic measurements and cardiac catheterization findings in all patients, and groups with impaired and preserved CFVR, respectively.

	All Patients (*n* = 48)	Impaired CFVR (*n* = 21)	Preserved CFVR (*n* = 27)	*p* Value
Aortic regurgitation > +1/4 (%)	20.0	15.0	24.0	0.71
Aorta, systolic pressure (mmHg, mean, SD)	110 ± 13	108 ± 13	111 ± 13	0.37
Aorta, diastolic pressure (mmHg, mean, SD)	68 ± 10	65 ± 8	70 ± 10	0.082
Aorta, mean pressure (mmHg, mean, SD)	85 ± 10	84 ± 10	86 ± 11	0.46
Left ventricular systolic pressure (mmHg, mean, SD)	114 ± 16	111 ± 17	115 ± 15	0.36
LV end-diastolic pressure (mmHg, mean, SD)	13 ± 5	12 ± 6	13 ± 5	0.52
Right atrial a wave (mmHg, mean, SD)	11 ± 4	11 ± 4	12 ± 4	0.77
Right atrial x wave (mmHg, mean, SD)	8 ± 3	8 ± 3	8 ± 4	0.77
Right atrial mean pressure (mmHg, mean, SD)	10 ± 4	10 ± 4	10 ± 4	0.87
Right ventricular systolic pressure (mmHg, mean, SD)	42 ± 9	44 ± 7	40 ± 10	0.26
Right ventricular end-diastolic pressure (mmHg, mean, SD)	10 ± 4	10 ± 5	9 ± 3	0.39
Left pulmonary artery branch stenosis (%)	21.0	35.0	8.3	0.051
Right pulmonary artery branch stenosis (%)	21.0	25.0	17.0	0.71
Pulmonary artery systolic pressure (mmHg, mean, SD)	33 ± 9	35 ± 7	30 ± 10	0.071
Pulmonary artery diastolic pressure (mmHg, mean, SD)	12 ± 5	12 ± 3	11 ± 5	0.33
Pulmonary artery mean pressure (mmHg, mean, SD)	19 ± 5	21 ± 5	18 ± 4	0.030
Pulmonary artery mean pressure > 20 mm Hg (%)	43.0	60.0	29.0	0.041

## Data Availability

The raw data supporting the conclusions of this article will be made available by the authors on request.

## Abbreviations

The following abbreviations are used in this manuscript:

ASO	Arterial Switch Operation
BMI	Body Mass Index
BSA	Body Surface Area
CFVR	Coronary Flow Velocity Reserve
CI	Confidence Interval
CMR	Cardiac Magnetic Resonance
CoAo	Coarctation of the Aorta
D-TGA	Dextro-Transposition of the Great Arteries
ECG	Electrocardiogram
GLS	Global Longitudinal Strain
LAD	Left Anterior Descending (coronary artery)
LV	Left Ventricle/Left Ventricular
LVEDD	Left Ventricular End-Diastolic Diameter
LVESD	Left Ventricular End-Systolic Diameter
LVEF	Left Ventricular Ejection Fraction
LVOTO	Left Ventricular Outflow Tract Obstruction
MAP	Mean Arterial Pressure
MAPSE	Mitral Annular Plane Systolic Excursion
mPAP	Mean Pulmonary Artery Pressure
MSCT	Multi-slice Computed Tomography
NTproBNP	N-terminal pro-B-type Natriuretic Peptide
OR	Odds Ratio
PGE1	Prostaglandin E1
RA	Right Atrium
RV	Right Ventricle/Right Ventricular
RVSP	Right Ventricular Systolic Pressure
SF	Shortening Fraction
STE	Speckle Tracking Echocardiography
TAPSE	Tricuspid Annular Plane Systolic Excursion
TV	Tricuspid Valve
VSD	Ventricular Septal Defect
